# Enhancement of the ammonia synthesis activity of a Cs- or Ba-promoted ruthenium catalyst supported on barium niobate

**DOI:** 10.1039/d4ra03301a

**Published:** 2024-06-10

**Authors:** Minxuan Chen, Qiang Zhang, Zhixiong You

**Affiliations:** a Eco-Environmental Damage Judicial Expertise Center, Hubei Provincial Academy of Eco-Environmental Sciences Wuhan 430079 PR China; b Hubei Key Laboratory of Pollutant Damage Assessment and Environmental Health Risk Prevention and Control Wuhan 430079 PR China; c School of Resources and Environmental Sciences, Wuhan University Wuhan 430079 PR China

## Abstract

Barium niobates with different crystalline structures and morphologies were prepared *via* a hydrothermal method and applied as a support for a ruthenium catalyst in ammonia synthesis. The sample synthesized with a nominal Ba/Nb ratio = 2.0, having a pure Ba_5_Nb_4_O_15_ crystalline phase and uniform flake-like structure, exhibited the best performance as a support in ammonia synthesis. The flake-like substrate favored the uniform distribution of ruthenium on its surface, which could promote ruthenium to expose more B5 sites. Addition of a Ba- or Cs-promoter enhanced the activity of the Ru/Ba_5_Nb_4_O_15_ catalyst markedly. The highest rate of ammonia synthesis over 2Cs- and 1Ba-4 wt% Ru/Ba_5_Nb_4_O_15_ was 4900 and 3720 (μmol g^−1^_cat_ h^−1^) at 0.1 MPa and 623 K, respectively. Both catalysts were stable during the reaction for 72 h at 673 K and 0.1 MPa. Thus, the synthesized Ba_5_Nb_4_O_15_ is expected to be a promising oxide support for ruthenium catalysts for ammonia synthesis.

## Abstract 2

Ba- or Cs–Ru/Ba_5_Nb_4_O_15_ catalysts demonstrated a significant improvement of the ammonia synthesis rate.

## Graphical abstract

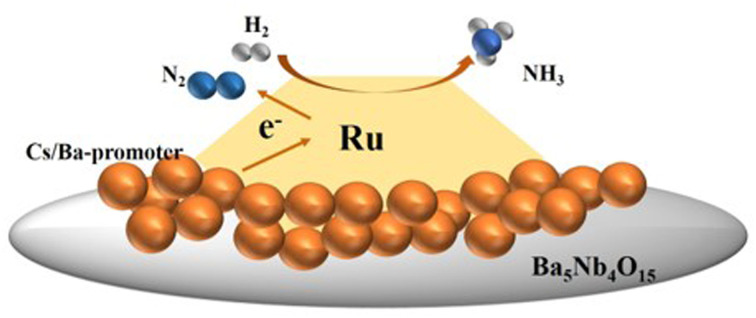

## 1. Introduction

Ammonia plays a very important role in the chemical industry and is often used in fields such as fertilizers and pharmaceutical intermediates. In recent years, liquid ammonia as a hydrogen-storage material has received widespread attention. Industrial synthesis of ammonia mainly adopts the Haber–Bosch process under high temperature and pressure, and often uses iron-based catalysts, which causes high energy consumption and considerable pollution.^1^ Therefore, developing catalysts that can achieve a high performance at low temperature and low pressure is the key to this process.

In the Haber–Bosch process, ruthenium-based catalysts exhibit high activity for ammonia synthesis under mild conditions, making them the most promising second-generation catalysts to replace industrial iron-based catalysts.^2–4^ The ruthenium-base catalyst requires a suitable support to disperse and support the ruthenium component. At present, electronic compounds,^5–7^ hydrides,^8^ carbon materials^9–11^ and metal oxides^12,13^ are used as supports of ruthenium-based catalysts. Li *et al.*^14^ investigated Nvs-g-C_3_N_4_/graphene as a support. Related studies have shown that electronic compounds and hydrides have good electron-transfer capabilities, but their preparation is complex and difficult to put into practical application. Carbon materials have a large specific surface area but are prone to methanation.^15^ Metal oxides have attracted the attention of researchers due to their stable structure, simple synthesis, good conductivity and thermal stability. In addition to traditional metal oxides such as MgO^16–18^ and Al_2_O_3_,^19–21^ special structures such as BaCeO_3_,^22^ Sr_2_Nb_2_O_7_,^23^ Mo_2_TiC_2_O_2_ (ref. 24) and MgAl-LDO (ref. 25) have been reported in recent years.

The introduction of promoters can improve the dispersion of Ru, increase the electron density on the surface of Ru, reduce the activation energy of dissociated N_2_, and thereby enhance the activity of ammonia synthesis.^26,27^ There are various forms and types of additives, among which some alkali metals (K, Cs) and alkali-earth metals (Ba) are widely studied due to their lower electronegativity, which is more conducive to electron transfer to the surface of Ru and promotes the ammonia synthesis reaction.^28–31^

We have studied Sr_2_Nb_2_O_7_ and Sr_2_Ta_2_O_7_ as supports for ruthenium catalysts, which can exhibit high activity at milder reaction conditions. Numerous studies have shown a strong interaction between alkaline oxide carriers and Ru, making them ideal metal-oxide carriers. Ba and Sr belong to the same group of elements, and Ba has stronger alkalinity. Herein, we demonstrate that Ba- or Cs-promoted Ru supported on a barium niobate substrate exhibited superior activity.

## 2. Materials and methods

### 2.1 Preparation of the Ba_5_Nb_4_O_15_ support

The Ba_5_Nb_4_O_15_ support was prepared by a hydrothermal method. First, a precursor of niobium (Nb_2_O_5_·*n*H_2_O) was synthesized from NbCl_5_*via* a hydrothermal method according to a published report.^32^ An alcohol solution of NbCl_5_ (0.37 mol L^−1^) was prepared by dissolving an appropriate amount of NbCl_5_ (99.0%; MilliporeSigma) in ethanol (analytical purity; Sinopharm Chemical Reagents). Then, the pH of the solution was adjusted to ∼10.5 by adding NH_4_OH aqueous solution (4 wt%) with stirring at room temperature for 4 h. Then, a Teflon™-lined autoclave was used to hold this mixture, followed by treatment at 473 K for 24 h. Subsequently, a solid product was recovered by centrifugation (8000 rpm for 30 min) and washed repeatedly with pure water until the filtrate was confirmed to be free of chloride anions using AgNO_3_. Finally, the product was dried at 353 K under a vacuum of ∼1.0 kPa.

Second, Ba(OH)_2_·8H_2_O (99.5%; MilliporeSigma) and the resultant Nb_2_O_5_·*n*H_2_O was dispersed in 45 mL of pure water and stirred for 0.5 h at room temperature. Then, a Teflon-lined autoclave was used to hold this mixture, followed by treatment at 473 K for 24 h. The resultant product was filtered and washed with pure water to near neutral pH. The product was dried in a vacuum (∼1.0 kPa) at 353 K for 10 h.

### 2.2 Preparation of the supported Ru catalyst

Ruthenium catalysts were synthesized by an impregnation method. A certain amount of Ru_3_(CO)_12_ (99.0%; MilliporeSigma) was dissolved in approximately 10–25 mL of tetrahydrofuran (THF) solution. Then, dried Ba_5_Nb_4_O_15_ (0.20 g) was added. The mixture was stirred at room temperature for 12 h. Then, the solvent was removed using a rotary evaporator (313 K, 10 kPa). Dried samples were decomposed at 723 K for 3 h in an Ar (99.999%) flow of 5 mL min^−1^. The catalysts obtained had a nominal Ru loading of 4 wt%. An appropriate amount of CsNO_3_ or Ba(NO_3_)_2_ was added to the obtained Cs- or Ba- Ru/Ba_5_Nb_4_O_15_ catalyst by impregnation.

### 2.3 Activity measurements

The activity of the Cs- or Ba- Ru/Ba_5_Nb_4_O_15_ catalysts for ammonia synthesis was tested in a fixed-bed plug-flow reaction system. Prior to tests, the dried catalyst samples were ground, pelletized, crushed and sieved. Then, the pellets (0.1000 g) of size 0.22–0.45 mm were loaded into a quartz tubular reactor (I.D. = 7 mm). Before ammonia synthesis, the catalysts were treated in H_2_ (99.999%) flow at 573–873 K for 3 h to reduce ruthenium cations to a metallic state and to decompose CsNO_3_ or Ba(NO_3_)_2_. Ammonia synthesis reactions were carried out at 573 to 773 K and 0.10 MPa in a synthesis gas flow (H_2_/N_2_ = 3/1, 99.999%, 60 mL min^−1^). The rate of ammonia formation was calculated based on the rate of decrease of the conductivity of H_2_SO_4_ solution. During activity tests, after the product gas had been stabilized under each reaction condition for 30 min, it was introduced into a sulfuric acid solution for measurement.

### 2.4 Characterization

The crystalline structure of samples was analyzed by X-ray diffraction (XRD) performed on an X'Pert Pro diffractometer (PANalytical) equipped with a Cu-Kα radiation source (*λ* = 1.5405 Å). All diffraction patterns were recorded in a 2*θ* range of 10–70° at a scan speed of 2° min^−1^.

The data for the N_2_ adsorption–desorption isotherms collected at 77.3 K on an apparatus for automatic measurement of specific surface area and pore-size distribution (BELSORP-mini II; Microtrac) were applied for evaluating the Brunauer–Emmett–Teller (BET) surface area of the prepared samples. The samples were degassed at 473 K under 10^−2^ kPa for 2 h before the tests. The specific surface area was calculated from the linear part of the BET plot, where the *P*/*P*_0_ ratios were 0.05–0.25.

The content of Nb and Ba in the products was detected by inductively coupled plasma-atomic emission spectroscopy (ICP-AES) using the IRIS Intrepid II XSP system (Thermo Fisher Scientific).

The morphology of synthesized Ba_5_Nb_4_O_15_ samples was observed by scanning electron microscopy (SEM) using a Zeiss system (Sigma) at an acceleration voltage of 20 kV. Images of Ru nanocrystallites on supports were recorded by transmission electron microscopy (TEM) on a JEM-2100 system (JEOL) at an acceleration voltage of 200 kV.

Temperature programmed desorption (TPD) tests were carried out on a self-built device, which was connected to a gas chromatograph (GC-2020; Trustworthy) equipped with a thermal conductivity detector (TCD). Before the desorption test, the catalyst (100.0 mg) was pretreated in N_2_ (99.999%) flow of 15 mL min^−1^ at 773 K for 3 h, cooled down to 373 K, and exposed to a flow of 5 vol% NH_3_ (balanced by 99.999% N_2_) of 10 mL min^−1^ for 30 min to allow the sample to reach an adsorption equilibrium. Then, N_2_ (99.999%) flow of 15 mL min^−1^ at 373 K for 30 min was used to remove the physically adsorbed ammonia molecules. In the desorption test, the catalyst was heated from 373 to 1073 K at 10 K min^−1^. The desorbed ammonia was carried in N_2_ flow (15 mL min^−1^) into the gas chromatograph for quantification.

## 3. Results and discussion

### 3.1 Effect of the Ba/Nb molar ratio on the morphology of barium-niobate samples

**Fig. 1:**
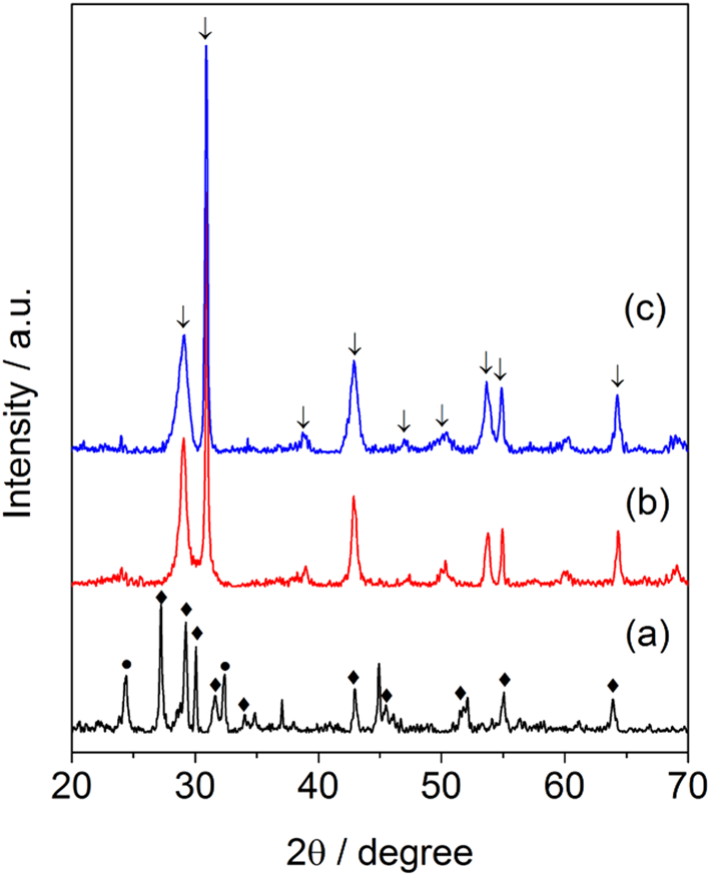
XRD patterns of barium-niobate samples prepared by a hydrothermal reaction at 473 K for 24 h with different molar ratios of Ba/Nb. (a) Ba/Nb = 0.5, (b) Ba/Nb = 2.0, (c) Ba/Nb = 4.0.

Fig. 1 displays the XRD patterns of the samples of synthesized barium niobate. It can be seen that the molar ratio of Ba(OH)_2_/Nb_2_O_5_ affected the formation of a crystalline structure of obtained materials. Ba_4_Nb_2_O_9_ and Nb_2_O_5_ phases (Fig. 1a) were observed in the sample while an equal amount of Ba(OH)_2_ and Nb_2_O_5_ (Ba/Nb = 0.5) was mixed and hydrothermally treated. By increasing Ba/Nb to 2.0, the other phases disappeared (Fig. 1b) and we could obtain a sample containing Ba_5_Nb_4_O_15_ mainly in the orthorhombic phase (PDF#14-0028). Increasing Ba/Nb to 4.0 led to no apparent change in the crystalline structure of the samples (Fig. 1c). It can be inferred that the added Ba(OH)_2_ exceeding the stoichiometry of Ba_5_Nb_4_O_15_ had almost no impact on the crystalline structure of the resultant barium niobates. ICP-AES (Table 1) confirmed the XRD results that the measured Ba/Nb values agreed well with nominal Ba/Nb (added Ba/Nb) when the Ba/Nb ratio = 1. For Ba/Nb = 2 and 4, the measured Ba/Nb values were close to 1.2, which was consistent with the XRD pattern. These results indicated that excessive addition of Ba(OH)_2_ was lost during filtration and water-washing after hydrothermal treatment.

**Table 1:** ICP-OES results of Nb and Ba content in different Ba/Nb hydrothermal products at 473 K for 24 h

Property		
Sample weight (g)	Added Ba/Nb	Measured Ba/Nb (mole ratio)
0.0103	1	1.01
0.0110	1.25	1.10
0.0106	2	1.11
0.0102	4	1.20

**Table 2:** Ammonia adsorbed by Ba_5_Nb_4_O_15_ (2.0 BaNb), Sr_2_Nb_2_O_7_ (2.0 SrNb), γ-Al_2_O_3_, and MgO

Support	BET surface area (m^2^ g^−1^)	Adsorbed NH_3_	
		(μmol g^−1^)	(μmol m^−2^)
Sr_2_Nb_2_O_7_	83	122.3	0.13
Ba_5_Nb_4_O_15_	19	30.4	0.15
Al_2_O_3_	85	236.2	0.28
MgO	14	74.3	0.54

Corresponding to their crystalline structures, elongated crystalline grains of size 200 × 1000 nm (Fig. 3a) could be obtained when the sample was prepared with a nominal Ba/Nb = 1. Increasing the addition amount of Ba(OH)_2_ to Ba/Nb ≥2.0, we obtained Ba_5_Nb_4_O_15_ flakes, and the size of the flakes decreased slightly with increasing the nominal Ba/Nb (Fig. 3b and c). These results indicated that excessive addition of Ba(OH)_2_ accelerated the nucleation rate of niobates in hydrothermal synthesis and, as a result, aggregates of flakes were formed.

**Fig. 2:**
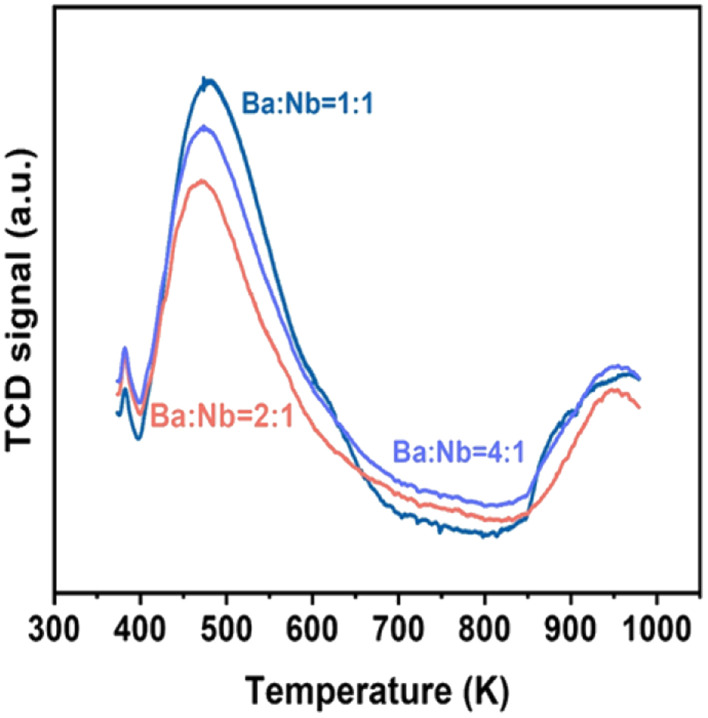
Ammonia TPD profiles obtained over different molar ratios of Ba/Nb. Samples (100.0 mg) were exposed to 5 vol% NH_3_ in N_2_ flow (10 mL min^−1^) at 373 K for 30 min, and desorptions were performed from 373 to 1073 K at 10 K min^−1^ in N_2_ carrier gas flow (15 mL).

**Fig. 3:**
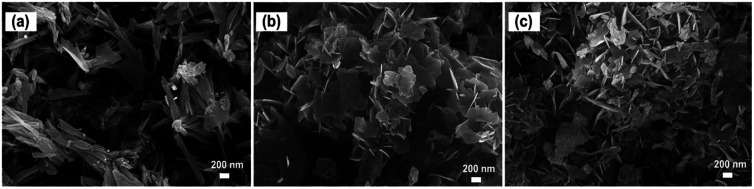
SEM images of barium-niobate samples prepared by a hydrothermal reaction at 473 K for 24 h with different molar ratios of Ba/Nb: (a) Ba/Nb = 1.0, (b) Ba/Nb = 2.0, (c) Ba/Nb = 4.0.

### 3.2 Catalytic activity of Ru/Ba_5_Nb_4_O_15_

**Fig. 4:**
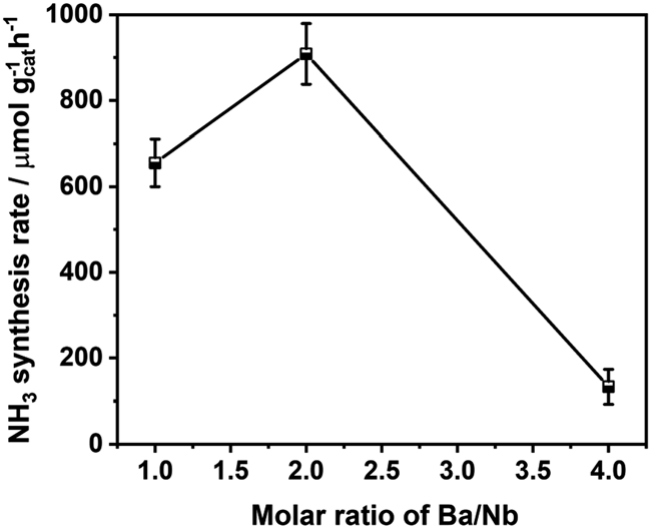
Ammonia synthesis rate over a 4 wt% Ru catalyst supported on barium niobates prepared with different nominal molar ratios of Ba/Nb. Reaction conditions: prior to activity tests, the catalysts (100.0 mg) were reduced in H_2_ flow of 15 mL min^−1^ at 573 K for 3 h; the activities were tested in 60 mL (STP) min^−1^ of the synthesis gas (3H_2_ + N_2_, 99.999%) at 673 K and 0.1 MPa.

Fig. 4 compares the ammonia synthesis rates over 4 wt% Ru catalysts supported on barium niobates synthesized with different nominal molar ratios of Ba/Nb at 673 K and 0.1 MPa. The 4 wt% Ru/Ba_5_Nb_4_O_15_ catalyst prepared with a nominal Ba/Nb = 2.0 gave the highest activity of 909 μ mol g^−1^_cat_ h^−1^. Further increase in the nominal Ba/Nb to >2.0 reduced the activity slightly (Fig. 4). Therefore, we chose Ba_5_Nb_4_O_15_ prepared at Ba/Nb = 2.0 as the support for Ru catalysts to study the effect of promoters. The acidity of the oxide support is detrimental to the activity of Ru catalysts for ammonia synthesis, and the surface acidity can be reflected by TPD. The TPD profile (Fig. 2) suggested that the acid sites were weaker on a catalyst prepared with a nominal Ba/Nb = 2.0 than other catalysts, which was consistent with the results of the activity test.

**Fig. 5:**
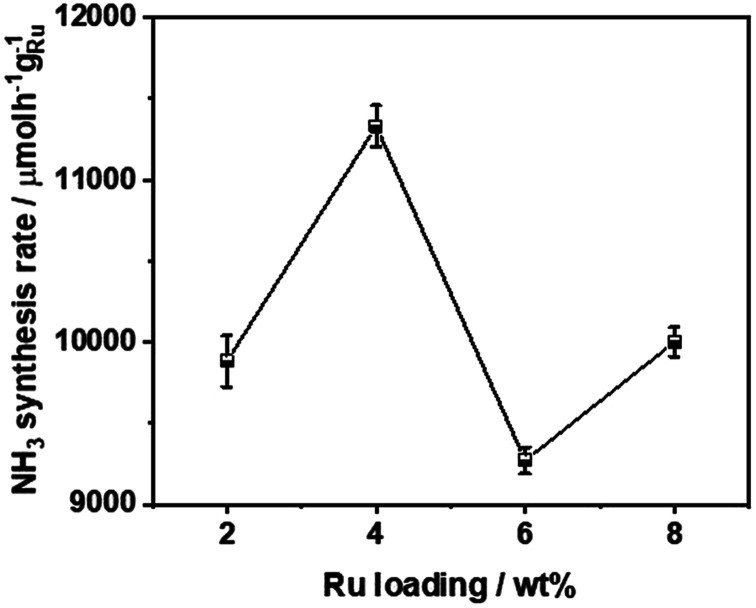
Ammonia synthesis rate of different Ru loadings on Ba_5_Nb_4_O_15_. Reaction conditions: prior to activity tests, the catalysts (100.0 mg) were reduced in H_2_ flow of 15 mL min^−1^ at 573 K for 3 h; the activities were tested in 60 mL (STP) min^−1^ of synthesis gas (3H_2_ + N_2_, 99.999%) at 673 K and 0.1 MPa.

The ammonia synthesis rate of different Ru loadings on Ba_5_Nb_4_O_15_ is shown in Fig. 5. The-ammonia synthesis rate increased first and then decreased with an increase in the addition amount of Ru, and reached a peak at 4 wt% Ru loading. Therefore, we chose 4 wt% Ru/Ba_5_Nb_4_O_15_ to study the effect of promoters.

**Fig. 6:**
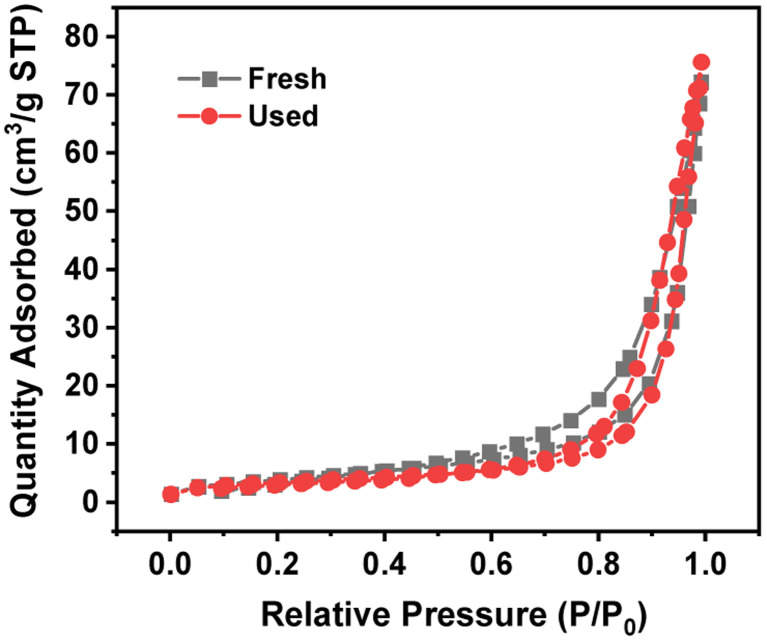
Adsorption/desorption isotherm of fresh and used 4% Ru/Ba_5_Nb_4_O_15_.

Table 3 lists the BET specific surface area, total pore volume and pore width of 4 wt% Ru/Ba_5_Nb_4_O_15_. Fig. 6 shows the adsorption/desorption isotherms of fresh and used 4% Ru/Ba_5_Nb_4_O_15_. The BET specific surface area and pore volume of the fresh catalyst and used catalyst showed almost no change. The average pore size increased after the reaction, possibly due to the detachment of impurities caused by a high temperature.

**Table 3:** BET specific surface area, total pore volume (*V*_p_), and pore width of 4 wt% Ru/Ba_5_Nb_4_O_15_

Catalyst	*S*_BET_ (m^2^ g^−1^)	Pore volume (cm^3^ g^−1^)	Average pore width (nm)
Fresh	15	0.11	29.67
Used	13	0.11	38.32

### 3.3 Catalytic activity of Ba- or Cs-promoted Ru/Ba_5_Nb_4_O_15_

Tables 4 and 5 list the ammonia synthesis rates over 1Ba- and 2Cs-4 wt% Ru/Ba_5_Nb_4_O_15_ activated in the synthesis gas (3H_2_ + N_2_) flow at different temperatures for 3 h. For both catalysts, the ammonia synthesis rate first increased and then decreased when increasing the activation temperature. The optimal activation temperature for Ba- and Cs-promoted catalysts was 773 and 673 K, respectively. The activation process could transform the promoter into the oxide and/or hydroxide form. In addition, it could form and grow the Ru nanoparticle. A low activation temperature may not be sufficient to decompose the nitrate precursor of the promoter. However, sintering of supported Ru particles would occur and their particle size could increase at higher activation temperatures (*e.g.*, 500 °C). Therefore, too low or high activation temperatures are not conducive to the formation of high performance.

**Table 4:** Ammonia synthesis rate over 1Ba-4 wt% Ru/2.0BaNb activated at different temperatures in the synthesis gas (3H_2_ + N_2_, 60 mL (STP) min^−1^) for 3 h

Activation temperature (K)	NH_3_ synthesis rate at 0.1 MPa (μmol g^−1^_cat_ h^−1^)		
	623 K	673 K	723 K
673	126	800	3196
773	1936	3720	2471
873	93	630	3062

**Table 5:** Ammonia synthesis rate over 2Cs-4 wt% Ru/2.0BaNb activated at different temperatures in the synthesis gas (3H_2_ + N_2_, 60 mL (STP) min^−1^) for 3 h

Activation temperature (K)	NH_3_ synthesis rate at 0.1 MPa (μmol g^−1^_cat_ h^−1^)		
	623 K	673 K	723 K
573	10	1416	4974
673	2363	4900	3283
773	0	560	2685

Table 6 details the catalytic activity of Ru-based catalysts promoted with Ba(NO_3_)_2_ and CsNO_3_. It can be seen that all the promoters promoted the catalytic performance of the Ru/Ba_5_Nb_4_O_15_ catalyst markedly for ammonia synthesis. Table 6 also compares the effect of different molar ratios of promoters. For the Ba-promoter, the ammonia synthesis rate was first improved when the molar ratio of Ba/Ru was raised from 0 to 1, and then decreased when the molar ratio increased to 2. Therefore, the optimal molar ratio of Ba/Ru was 1, corresponding to ammonia synthesis of 3720 μ mol g^−1^_cat_ h^−1^ at 400 °C and 0.1 MPa. For the Cs-promoter, a molar ratio of Cs/Ru at 1 and 2 could both exhibit preferable activity for ammonia synthesis, but 2Cs/Ru could elicit higher activity at milder reaction conditions, and ammonia synthesis was 2363 and 4900 μ mol g^−1^_cat_ h^−1^ at 573 K and 623 K, respectively. Both were 20- or 30-times higher than that of the unpromoted catalyst. Therefore, we postulated that 2Cs/Ru was the best loading ratio. A structural promoter can synthesize more B_5_ active sites.^33,34^ In the 1Ba-4 wt% Ru/Ba_5_Nb_4_O_15_ catalytic system, Ba is considered to be a structural promoter. This status leads to surface reconstruction of Ru and an increase in the number of B_5_ active sites, thereby improving the performance of the catalyst. In the 2Cs-4 wt% Ru/Ba_5_Nb_4_O_15_ catalytic system, Cs is considered to be an electronic promoter that can transfer electrons to the surface of active metals and reduce the dissociation energy of N_2_. In addition, barium in the carrier could have a synergistic effect with the Cs-promoter to improve the activity of ammonia synthesis significantly.

**Table 6:** Ammonia synthesis rate over 4 wt% Ru/2.0BaNb (Ba_5_Nb_4_O_15_) catalysts promoted by barium or cesium^a^

Catalyst	NH_3_ synthesis rate (μmol g^−1^_cat_ h^−1^)			
	573 K	623 K	673 K	723 K
Ru/BN	64	164	453	1106
Ba(0.5)–Ru/BN	519	2607	3262	2084
Ba(1)–Ru/BN	1936	3720	2471	1371
Ba(2)–Ru/BN	343	1743	3331	2754
Cs(1)–Ru/BN	1404	5048	3896	2413
Cs(2)–Ru/BN	2363	4900	3283	1773
Cs(4)–Ru/BN	1128	3454	2704	1523

a Conditions for ammonia synthesis: 100.0 mg of catalyst, 60 mL (STP) min^−1^ of synthesis gas (3H_2_ + N_2_, 99.999%) at 0.1 MPa. Prior to activity tests, catalysts were activated at 573 K for 3 h in 60 mL (STP) min^−1^ the synthesis gas (3H_2_ + N_2_, 99.999%). Each rate of ammonia synthesis was determined over 30 min after 30 min stabilization under given conditions. Values before the promoter in the catalyst names are the molar ratios of Ba or Cs to Ru.

**Fig. 7:**
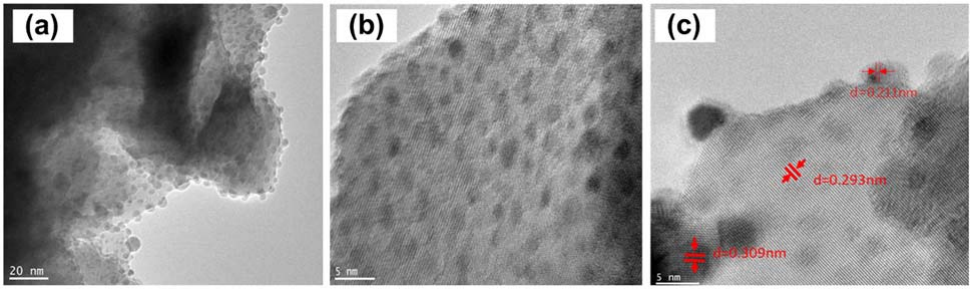
TEM images of (a)–(c) 4 wt% Ru/2.0BaNb prior to TEM observations. Catalysts were reduced in the synthesis gas (3H_2_ + N_2_, 60 mL (STP) min^−1^) at their corresponding activation temperature (573 K).

Fig. 7 shows the morphology of Ru nanoparticles on Ba_5_Nb_4_O_15_ substrate after reduction by exposure to H_2_ and N_2_ at 573 K for 3 h. Ru nanoparticles were evenly distributed on the surface of the Ba_5_Nb_4_O_15_ support. The HRTEM image (Fig. 7b) shows hexagonal ruthenium particles uniformly deposited on the substrate. The lattice spacing of Ru particles was 0.211 nm, which corresponds to the (101) planes of Ru crystals.^35,36^ The lattice spacing of 0.293 and 0.309 nm corresponded to the (103) planes of Ru crystals and (110) planes of Ba_5_Nb_4_O_15_ crystals, respectively.^37,38^ From the XRD spectrum of Ba_5_Nb_4_O_15_ synthesized with Ba/Nb = 2.0, the (110) and (103) peaks were the top-two most intensive diffractions, indicating that they were the preferred exposed facets of the Ba_5_Nb_4_O_15_ flakes. These exposed facets, consisting of oxygen anions, can induce the epitaxial growth of Ru nanocrystallites.^39^ It was demonstrated that the density of B_5_ sites on the epitaxial growth of Ru was likely to be substantially greater than that for round particles. Fig. 7b reveals that the Ru particles seemed to be encapsulated, which explained why Ru/Ba_5_Nb_4_O_15_ did not adsorb H_2_ in the H_2_-pulse titration method. This phenomenon was attributed to the strong metal–support interaction (SMSI), which results in partial covering of Ru particles by the reduced support species.^40^

**Fig. 8:**
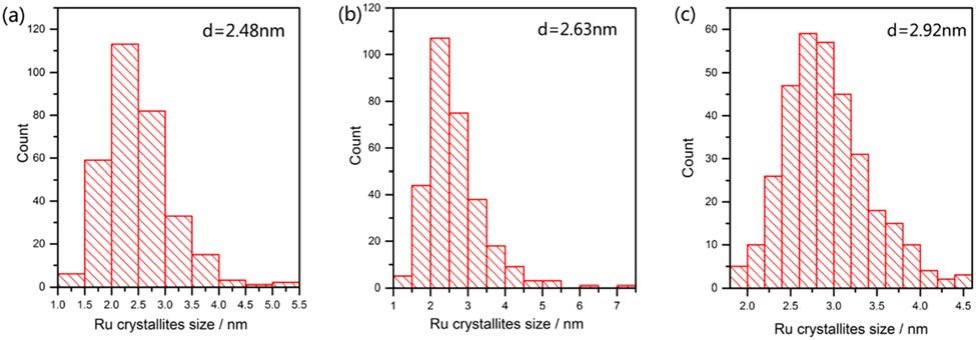
Size distribution of 4 wt% Ru particles supported on BaNb. Catalysts were reduced in the synthesis gas (3H_2_ + N_2_, 60 mL (STP) min^−1^) at activation temperatures of (a) 573 K, (b) 673 K, and (c) 773 K.

**Fig. 9:**
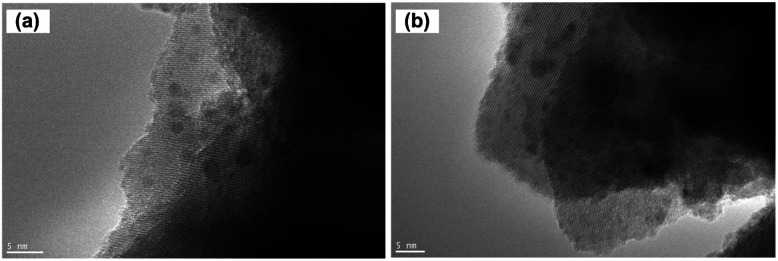
TEM images of (a) 1Ba-4 wt% Ru/2.0BaNb and (b) 2Cs-4 wt% Ru/2.0BaNb. Prior to TEM observations, catalysts were reduced in the synthesis gas (3H_2_ + N_2_, 60 mL (STP) min^−1^) at their corresponding activation temperature of 773 K (1Ba-4 wt% Ru/2.0BaNb) and 673 K (2Cs-4 wt% Ru/2.0BaNb) for 3 h.

In order to explore the effect of reduction temperature on the size of ruthenium nanoparticles, over 300 ruthenium particles were measured from TEM images. Fig. 8 shows the size distribution of Ru particles at 573, 673 and 773 K; the average particle size of ruthenium was 2.48, 2.63 and 2.92 nm, respectively. The average particle size of ruthenium became larger as the temperature increased. Due to cover by Ba- or Cs-promoters (Fig. 9a and b) at room temperature, much fewer Ru particles were observed. However, they presented comparable sizes with those of Ru catalysts on Ba_5_Nb_4_O_15_. These Ru particles became exposed to the reactants at ammonia synthesis conditions.^41,42^

**Fig. 10:**
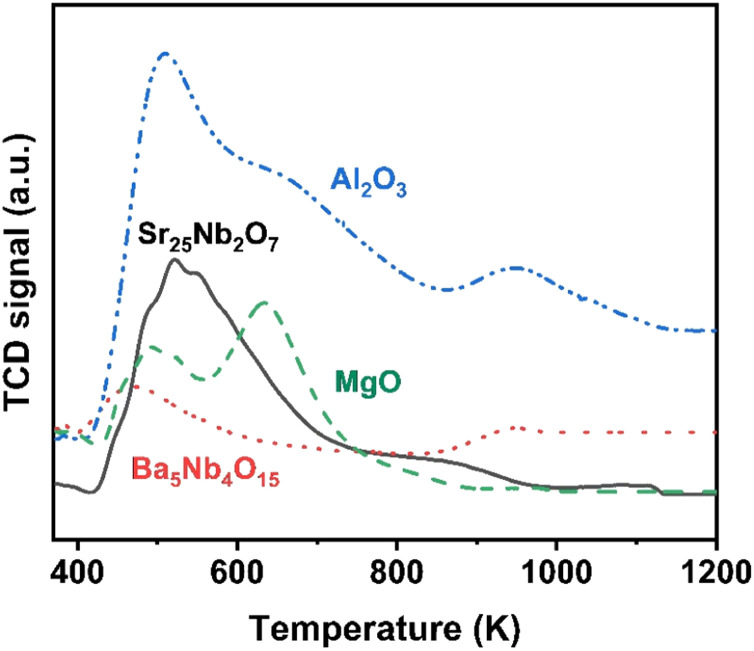
NH_3_-TPD profiles obtained over Ba_5_Nb_4_O_15_ (2.0 BaNb), Sr_2_Nb_2_O_7_ (2.0 SrNb), γ-Al_2_O_3_, and MgO. Samples (100.0 mg) were exposed to 5 vol% NH_3_ in N_2_ flow (10 mL min^−1^) at 373 K for 30 min, and desorptions were performed from 373 to 1073 K at 10 K min^−1^ in N_2_ carrier gas flow (15 mL min^−1^).

The surface acidity of Ba_5_Nb_4_O_15_, Sr_2_Nb_2_O_7_, γ-Al_2_O_3_ and MgO determined by the TPD of ammonia is shown in Fig. 10. The amount of chemisorbed ammonia on these supports is given in Table 2. The amount of adsorbed ammonia by weight of oxides followed the sequence of γ-Al_2_O_3_ ≫ Sr_2_Nb_2_O_7_ > MgO > Ba_5_Nb_4_O_15._ The acid strength of oxides, reflected by desorption temperatures, was also very different. The ammonia molecules bound on Ba_5_Nb_4_O_15_ desorbed at 400–600 K with the gentle peak around 480 K. The ammonia TPD profile of Sr_2_Nb_2_O_7_ showed a main peak at around 520 K. MgO over a similar temperature range split into two main peaks located at about 490 and 630 K. This result suggested that the acid sites were weaker on Ba_5_Nb_4_O_15_ than on MgO. γ-Al_2_O_3_ and Sr_2_Nb_2_O_7_ desorbed ammonia over a wide temperature range (400–1075 K), indicating that Ba_5_Nb_4_O_15_ had stronger electron-donating ability and could provide electrons to ruthenium to improve the rate of ammonia synthesis.

Table 7 compares the catalytic performance of Ba- or Cs–Ru/Ba_5_Nb_4_O_15_ catalyst with that of Ru catalyst supported on other substrates.^43–45^ Due to different Ru-loading, we calculated the ammonia synthesis rate based on Ru weight. The Ba- or Cs-promoted Ru/Ba_5_Nb_4_O_15_ catalyst exhibited a distinguished activity for ammonia synthesis, which was 10% higher than that of Cs–Ru/MgO, and 10-times higher than that of Ba–Ru/AC (industrial catalyst). Although Ru/C_12_A_7_:e−, Ru/La_2_Ce_2_O_7_, and Ru/CaCN catalysts show higher activity compared with our catalysts, the synthesis and reduction of mayenite electride are slightly difficult and, for the other two catalysts, the reaction conditions are stricter than those for Ru/Ba_5_Nb_4_O_15._

**Table 7:** Comparison of ammonia synthesis activity over Ru catalysts supported on different substrates

Catalysts	Ru loading (%)	Reaction condition	NH_3_ synthesis rate (μmol g^−1^_cat_ h^−1^)	NH_3_ synthesis rate (μmol g^−1^_Ru_ h^−1^)	Ref.
Ba–Ru/Ba_5_Nb_4_O_15_	4	623 K, 0.1 MPa	3720	93	This work
Cs–Ru/Ba_5_Nb_4_O_15_	4	623 K, 0.1 MPa	5048	126.2	This work
Ba–Ru/Sr_2_Nb_2_O_7_	2	623 K, 0.1 MPa	421	21	22
Cs–Ru/Sr_2_Nb_2_O_7_	2	623 K, 0.1 MPa	1060	53	22
Ba–Ru/AC	9.1	623 K, 0.1 MPa	2228	25	5
Cs–Ru/MgO	6	623 K, 0.1 MPa	3353	56	5
Ru/C_12_A_7_:e−	1.2	673 K, 0.1 MPa	2757	230	5
Ru/Pr_2_O_3_	5	673 K, 0.1 MPa	3600	72	41
Ru/La_2_Ce_2_O_7_	4	723 K, 10 MPa	5500	137.5	42
Ru/CaCN	4	673 K, 0.9 MPa	6500	162.5	43

### 3.4 Catalytic stability of Ba- or Cs–Ru/Ba_5_Nb_4_O_15_

**Fig. 11:**
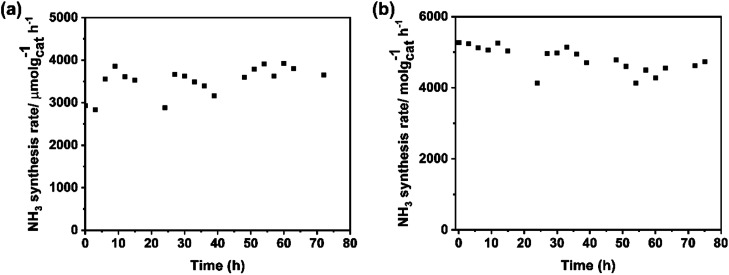
Ammonia synthesis rate over (a) 1Ba-4 wt% Ru/2.0BaNb and (b) 2Cs-4 wt% Ru/2.0BaNb as a function of time on steam. Reaction conditions: 100.0 mg of catalyst, 60 mL (STP) min^−1^ of synthesis gas (3H_2_ + N_2_) at 673 K and 0.1 MPa.

The stability of catalysts is the other important factor for practical application. The change in the rate of ammonia synthesis over Ba–Ru/Ba_5_Nb_4_O_15_ and Cs–Ru/Ba_5_Nb_4_O_15_ measured at 673 K and 0.1 MPa with time is shown in Fig. 11a and b. The ammonia synthesis rate showed no obvious change during 72 h reaction for the two catalysts, suggesting that the catalysts were stable.

The catalysts in three stages (*i.e.*, as-prepared (fresh), after 5 h activity tests (used) and after 72 h stability test (aged)) were analyzed by BET and XRD. After stability tests, the corresponding surface area of aged Ba–Ru/Ba_5_Nb_4_O_15_ and Cs–Ru/Ba_5_Nb_4_O_15_ was 15 and 13 m^2^ g^−1^, respectively, with a change in the surface area of <5% (Table 8). These results indicated that the textural morphology of the catalysts was stable under ammonia synthesis conditions.

**Table 8:** BET specific surface area of the 1Ba or 2Cs-4 wt% Ru/Ba_5_Nb_4_O_15_ catalyst measured at different phases

Catalyst	BET surface area (m^2^ g^−1^)		
	Fresh	Used	Aged
1Ba-4 wt% Ru/Ba_5_Nb_4_O_15_	18	16	15
2Cs-4 wt% Ru/Ba_5_Nb_4_O_15_	12	11	13

**Fig. 12:**
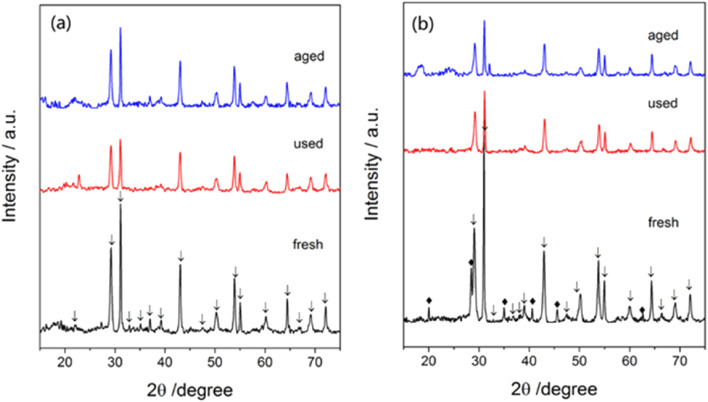
XRD images of (a) 1Ba–4Ru/Ba_5_Nb_4_O_15_ and (b) 2Cs–4Ru/Ba_5_Nb_4_O_15_ measured at different phases: as-prepared (fresh), after activity tests (used), and after stability tests (aged). Reaction conditions: 100.0 mg of catalyst, and 60 mL (STP) min^−1^ of synthesis gas (3H_2_ + N_2_), at 0.1 MPa.

A crystalline phase of Ba(NO_3_)_2_ (Fig. 12a) was not found but a crystalline phase of CsNO_3_ (Fig. 12b) was found in fresh catalysts. In contrast, no significant phase of CsNO_3_ was detected in used and aged catalysts, indicating the cesium promoter was present in an amorphous or well-dispersed state. After 72 h tests, the crystalline structure of the Ba_5_Nb_4_O_15_ substrate showed no obvious change, suggesting the catalysts to be of high performance and high stability for ammonia synthesis.

## 4. Conclusions

We demonstrated that flake-like Ba_5_Nb_4_O_15_ was an excellent support for an Ru catalyst for ammonia synthesis. Ba- or Cs-promoted Ru catalysts supported on Ba_5_Nb_4_O_15_ synthesized with a nominal Ba/Nb ratio = 2.0 exhibited high activity and stability for ammonia synthesis under mild reaction conditions compared to Cs–Ru/MgO (which is considered to be one of the most active oxide supports at present). Compared with other catalysts, Ba_5_Nb_4_O_15_ as a support exhibited higher activity at a lower temperature (623 K). Thus, barium niobate can be a promising candidate as a Ru catalyst for ammonia synthesis.

## Author contributions

Minxuan Chen: conceptualization, investigation, data analysis, and writing (original draft, review and editing). Zhixiong You: methodology, writing (review and editing), and data curation. Qiang Zhang: supervision and methodology.

## Conflicts of interest

The authors declare no conflict of interest.

## Supplementary Material

## References

[cit1] Zhang H., Wang L., Maréchal F. (2020). Appl. Energy.

[cit2] Kitano M., Inoue Y., Sasase M. (2018). Angew. Chem., Int. Ed..

[cit3] Zhou Y. P., Ma Y. C., Lan G. J. (2019). Chin. J. Catal..

[cit4] Kim S. Y., Lee H. W., Pai S. J. (2018). et al.. ACS Appl. Mater. Interfaces.

[cit5] Kitano M., Inoue Y., Yamazaki Y. (2012). Nat. Chem..

[cit6] Hattori M., Mori T., Arai T. (2018). ACS Catal..

[cit7] LI J., WU J., WANG H. (2019). et al.. Chem. Sci..

[cit8] Davis R. J. J. (2003). Catal.

[cit9] Ma Y. C., Lan G. J., Fu W. Z. (2020). J. Energy Chem..

[cit10] Nishi M., Chen S. Y., Takagi H. (2018). ChemCatChem.

[cit11] Lin B. Y., Guo Y. J., Cao C. F. (2018). Catal. Today.

[cit12] Lin B. Y., Heng L., Fang B. Y. (2019). ACS Catal..

[cit13] Ni J., Jing B. Q., Lin J. X. (2018). et al.. J. Rare Earths.

[cit14] Li D., Gao J., Hao M. (2023). Mol. Catal..

[cit15] Borisov V., Iost K., Temerev V. (2020). Diamond Relat. Mater..

[cit16] Aika K., Takano T., Murata S. (1992). J. Catal..

[cit17] Rosowski F., Hornung A., Hinrichsen O., Herein D., Muhler M., Ertl G. (1997). Appl. Catal., A.

[cit18] Muhler M., Rosowski F., Hinrichsen O., Hornung A., Ertl G. (1996). Stud. Surf. Sci. Catal..

[cit19] Aika K., Shimazaki K., Hattori Y., Ohya A., Ohshima S., Shirota K., Ozaki A. (1985). J. Catal..

[cit20] Aika K., Kumasaka M., Oma T., Kato O., Matsuda H., Watanabe N., Yamazaki K., Ozaki A., Onishi T. (1986). Appl. Catal..

[cit21] Miyazaki A., Balint I., Aika K., Nakano Y. (2001). J. Catal..

[cit22] Huang J., Yuan M., You Z. (2020). J. Catal..

[cit23] Chen M., Yuan M., You Z. (2018). Appl. Catal., A.

[cit24] Shu P., Qi X., Peng Q. (2023). MoCat.

[cit25] Marakatti S., Gaigneaux M. (2020). ChemCatChem.

[cit26] Kitano M., Inoue Y., Sasase M. (2018). Angew. Chem..

[cit27] Nishi M., Chen S., Takagi H. (2018). ChemCatChem.

[cit28] AIKA K.-I. (2017). Catal. Today.

[cit29] Lin B. Y., Guo Y. J., Cao C. F. (2018). Catal. Today.

[cit30] Davis R. J. (2003). J. Catal..

[cit31] Qiu J. Z., Hu J. B., Lan J. G. (2019). Chem. Mater..

[cit32] Fan W., Zhang Q., Deng W., Wang Y. (2013). Chem. Mater..

[cit33] Rarogpilecka W., Miskiewicz E., Szmigiel D., Kowalczyk Z. (2005). J. Catal..

[cit34] Huang J., Yuan M., Li X. (2021). Appl. Catal., A.

[cit35] Song Z., Cai T., Hanson J. C., Rodriguez J. A., Hrbek J. (2004). J. Am. Chem. Soc..

[cit36] Topsøe H., Wagner J. B., Hansen P. L., Dahl S., Hansen T. W., Jacobsen C. J. H. (2001). Science.

[cit37] Kehres J., Jakobsen J. G., Andreasen J. W. (2012). Phys. Chem. C..

[cit38] Wang K., Wu X. Y., Zhang G. K. (2018). Chem. Eng..

[cit39] Havelia S., Wang S., Schultz M. (2013). CrystEngComm.

[cit40] Feng J., Fu H., Wang J. (2008). Catal. Commun..

[cit41] You Z. X., Inazu K., Aika K., Baba T. (2007). J. Catal..

[cit42] Hansen T. W., Hansen P. L., Dahl S., Jacobsen C. J. H. (2002). Catal. Lett..

[cit43] Sato K., Imamura K., Kawano Y., Miyahara S., Yamamoto T., Matsumurac S., Nagaoka K. (2017). Chem. Sci..

[cit44] Han W., Li Z., Liu H. (2019). J. Rare Earths.

[cit45] Kishida K., Kitano M. (2020). ACS Appl. Energy Mater..

